# Hyponatraemia in imported malaria: the pathophysiological role of vasopressin

**DOI:** 10.1186/1475-2875-11-26

**Published:** 2012-01-26

**Authors:** Ewout J Hoorn, Marlies E van Wolfswinkel, Dennis A Hesselink, Yolanda B de Rijke, Rob Koelewijn, Jaap J van Hellemond, Perry JJ van Genderen

**Affiliations:** 1 Department of Nephrology, Erasmus MC, Rotterdam, The Netherlands; 2 Department of Internal Medicine, Harbour Hospital and Institute for Tropical Diseases, Haringvliet 72, 3011, TG Rotterdam, The Netherlands; 3 Department of Clinical Chemistry, Erasmus MC, Rotterdam, The Netherlands; 4 Laboratory for Parasitology, Harbour Hospital and Institute for Tropical Diseases, Rotterdam, The Netherlands; 5 Department of Medical Microbiology and Infectious Diseases, Erasmus MC, Rotterdam, The Netherlands

**Keywords:** Hyponatraemia, Malaria, Vasopressin, Copeptin, Falciparum, Import, AVP, SIADH

## Abstract

**Background:**

In the pathophysiology of hyponatraemia in malaria, the relative contribution of appropriate and inappropriate arginine vasopressin (AVP) release is unknown; the trigger for inappropriate AVP release is also unknown.

**Methods:**

Serum copeptin, a stable and sensitive marker for AVP release, was analysed in a large cohort of patients with imported malaria (204 patients) and in a small prospective substudy (23 patients) in which urine sodium and osmolality were also available. Hyponatraemia was classified as mild (serum sodium 131-134 mmol/l) and moderate-to-severe (< 131 mmol/l).

**Results:**

Serum copeptin on admission was higher in patients with moderate-to-severe hyponatraemia (median 18.5 pmol/L) compared with normonatraemic patients (12.7 pmol/L, *p *< 0.05). Despite prompt fluid resuscitation, the time to normalization of serum sodium was longer in patients with moderate-to-severe hyponatraemia (median 2.9 days) than in patients with mild hyponatraemia (median 1.7 days, *p *< 0.001). A poor correlation was found between serum sodium and copeptin levels on admission (r_s _= -0.17, *p *= 0.017). Stronger correlations were identified between serum C-reactive protein and copeptin (r_s _= -0.36, *p *< 0.0001) and between serum C-reactive protein and sodium (r_s _= 0.33, *p *< 0.0001). Data from the sub-study suggested inappropriate AVP release in seven of 13 hyponatraemic malaria patients; these patients had significantly higher body temperatures on admission.

**Conclusions:**

In hyponatraemic patients with imported malaria, AVP release was uniformly increased and was either appropriate or inappropriate. Although the exact trigger for inappropriate AVP release remains unknown, the higher body temperatures, correlations with C-reactive protein and long normalization times of serum sodium, suggest an important role of the host inflammatory response to the invading malaria parasite.

## Background

Hyponatraemia is a common finding in imported malaria and associated with severe *Plasmodium falciparum *malaria [1]. Nevertheless, its pathophysiology remains incompletely understood. Hyponatraemia is primarily a water balance disorder and usually caused by increased secretion of arginine vasopressin (AVP). With regard to hyponatraemia in malaria, some studies found evidence for "appropriate" vasopressin release due to hypovolaemia [2] whereas other studies found evidence for "inappropriate" vasopressin release [3,4]. There is, however, no consensus regarding the relative contributions of these mechanisms in the pathophysiology of hyponatraemia in malaria.

AVP is a key hormone in maintaining fluid balance and vascular tone [5]. Despite these important physiological functions, measurement of mature AVP is difficult and subject to preanalytical and analytical errors [6]. Recently, copeptin, a 39-amino acid glycopeptide that comprises the C-terminal moiety of the AVP precursor (CT-proAVP) was demonstrated to be a stable and sensitive marker for AVP release [6-9]. Furthermore, a number of studies have now shown that measurement of serum copeptin or calculation of the serum copeptin to urine sodium ratio is useful in the differential diagnosis of fluid and electrolyte disorders [10,11]. In the present study, serum copeptin levels were evaluated in a large cohort of patients with imported malaria to further explore the role of AVP in the pathophysiology of hyponatraemia in malaria.

## Methods

### Patients

The Harbour Hospital is a 161-bed general hospital located in Rotterdam, The Netherlands. It also harbours the Institute for Tropical Diseases, which serves as a national referral centre. The Rotterdam Malaria Cohort consists of all patients diagnosed with malaria at the Institute for Tropical Diseases in Rotterdam. In the period 1999-2010 the Rotterdam Malaria Cohort comprised 519 cases of imported malaria. Of all patients, anonymized demographic, clinical and laboratory data are routinely collected and stored in an electronic database. Moreover, in a large number of patients serum samples taken on admission were stored. For the present study, anonymized data from patients who entered the Rotterdam Malaria Cohort between January 1999 and December 2010 were used to estimate the prevalence of hyponatraemia in imported malaria at time of first presentation as well as for the follow-up of sodium levels after treatment during admission. For those patients with stored serum samples, copeptin levels were measured retrospectively. In a small sub-study serum copeptin levels were measured prospectively in addition to urinary sodium and osmolality.

### Laboratory investigations

All available laboratory data were measured on admission with the use of routine procedures. In contrast, copeptin levels were retrospectively measured in stored serum samples with a commercial sandwich immunoluminometric assay (Brahms Copeptin, Thermo Fisher Scientific, Hennigsdorf/Berlin, Germany) as described [9]. Normal values for serum copeptin in healthy volunteers range between 1.70 and 11.25 pmol/L [9]. Blood smears (thin and thick films) were obtained from finger pricks and stained with Giemsa for parasite counts. Malaria was diagnosed by Quantitative Buffy Coat analysis, *P. falciparum *Histidine-Rich-Protein 2 screening (now ICT Malaria, Binax) and conventional microscopy with subsequent specification of the *Plasmodium *species.

### Definitions

#### Severe malaria

Patients were considered as having severe *P. falciparum *malaria if they met the recently updated World Health Organization (WHO) criteria for severe malaria on admission or during hospitalization [12]. These criteria differ from the preset criteria [13] that were used to define severe malaria in previous studies [1].

#### Coma acidosis malaria (CAM) score

Of each patient with severe disease an admission CAM score, a 5-point (0-4 points) score calculated as the sum of the base deficit score (0-2 points) and Glasgow Coma score (0-2 points), was given [14].

#### Hyponatraemia

Hyponatraemia was defined as a serum sodium concentration of less than 135 mmol/L. Mild hyponatraemia was defined as a serum sodium concentration 131-134 mmol/L, whereas moderate hyponatraemia was defined as a serum sodium concentration 125-130 mmol/L. The threshold of < 131 mmol/Lwas chosen because a previous study found that a sodium level below 131 mmol/L was an independent predictor for severe disease in imported malaria [1]. Severe hyponatraemia was defined as a serum sodium level below 125 mmol/L. Given the low number of samples of patients with severe hyponatraemia in the copeptin study, these patients were grouped with the patients with moderate hyponatraemia to form the moderate-to-severe hyponatraemia group.

#### Inappropriate vs appropriate AVP secretion

A recent study found that the serum copeptin to urine sodium ratio may be used to differentiate normovolaemic hyponatraemia (ratio ≤ 30 pmol/mmol) from hypovolaemic hyponatraemia (ratio > 30 pmol/mmol) [10]. The most common example of normovolaemic hyponatraemia is the syndrome of inappropriate antidiuresis [15] and, therefore, a ratio ≤ 30 pmol/mmol was used to define inappropriate AVP release. Conversely, AVP release during hypovolaemic hyponatraemia is considered "appropriate" and, therefore, appropriate AVP release was defined as a ratio > 30 pmol/mmol.

### Statistical analysis

All data are reported as medians (range). Univariate comparisons were performed using the Kruskall-Wallis test (three groups) with Dunn's post-hoc tests, or the Mann-Whitney test (two groups) for not normally distributed data. Normally distributed data were compared with unpaired t-tests or unpaired t-tests with Welch correction, as appropriate. Correlations were analysed using Spearman rho (r_s_) and Wilcoxon's signed rank test. Kaplan-Meier survival curves for resolution of hyponatraemia after treatment were analysed with the Mantel-Cox log-rank test.

## Results

### Prevalence of hyponatraemia in imported malaria and its distribution among the various plasmodium species

Of the 519 cases in the Rotterdam Malaria Cohort 1999-2010, 10 (1.9%) patients had a severe hyponatraemia on admission, 60 (11.6%) patients had moderate hyponatraemia, whereas 166 (32.0%) malaria patients had mild hyponatraemia on admission, respectively. In the remaining 283 (54.5%) patients the sodium level on admission was normal. Of the 54 *P. falciparum *malaria patients fulfilling the criteria for severe disease, 5 (9.3%) patients had a severe hyponatraemia on admission, 20 (37.0%) patients had moderate hyponatraemia, whereas hyponatraemia was mild in 18 (33.3%) patients with severe malaria. Eleven (20.4%) patients with severe malaria had a normal sodium on admission, including the two patients who died. Of the 312 patients with uncomplicated *P. falciparum *malaria, severe hyponatraemia was present on admission in 4 (1.3%) patients, moderate hyponatraemia in 33 (10.6%) patients and a mild hyponatraemia was found in 105 (33.7%) patients on admission, respectively. Serum sodium concentrations were normal in the remaining 170 (54.5%) patients with uncomplicated *P. falciparum *malaria. Of the 153 patients with non-*P. falciparum *malaria, severe hyponatraemia was present in 1 (0.7%) patient, moderate hyponatraemia in 7 (4.6%) patients, mild hyponatraemia in 43 (28.1%) patients, and 102 (66.7%) patients had normal serum sodium concentrations on admission, respectively.

### Characteristics of the patients with severe malaria

When focusing on the 54 patients with severe malaria in the Rotterdam Malaria Cohort, these patients presented with the following severity criteria: hypotension (n = 1); impaired consciousness (n = 8) or unrousable coma characterized by a GCS ≤9 (n = 3); severe anaemia characterized by a haemoglobin level ≤3.0 mmol/L (n = 2) or a packed cell volume < 0.20 (n = 6); blackwater fever (n = 1); renal impairment characterized by a creatinine level ≥ 265 μmol/L (n = 6); liver impairment characterized by a total bilirubin level ≥ 50 mmol/L (n = 29); hyperlactataemia characterized by a lactate ≥ 5 mmol/L (n = 6); hyperparasitaemia characterized by a parasite load ≥ 5% (n = 34; on admission to the intensive care unit n = 40) and schizontaemia (n = 27), respectively. Of 30 patients with severe malaria a CAM score could be calculated on admission. The median CAM score was 1, and the scores ranged from 0 to 3. Nine patients had a CAM score of 0, 16 patients a CAM score of 1, four patients had a CAM score of 2, whereas 1 patient had a CAM score of 3.

Thirty-six patients received intravenous treatment with quinine, 11 patients were treated with intravenous artesunate. Four patients were solely treated with oral anti-malarials and the treatment mode was unknown in three patients. Thirty-two patients received exchange transfusion. Details of this adjunct therapy for severe malaria are published elsewhere [16]. Sixteen of 54 patients were referred from surrounding hospitals. There were no statistically significant differences between blood glucose levels (glucose 6.3 ± 1.7 mmol/L *vs *7.8 ± 5.0 mmol/L, *p *= 0.1099) and serum sodium levels on admission (sodium 133 ± 7 mmol/L *vs *130 ± 4 mmol/L, *p *= 02491) between patients referred from other hospital (n = 16) and those patients directly referred to the Institute for Tropical Diseases (n = 38), making a significant contribution of dextrose or glucose infusion on serum sodium levels on admission unlikely in the referred patients.

### Follow-up of hyponatraemia during hospitalization

**Figure 1:**
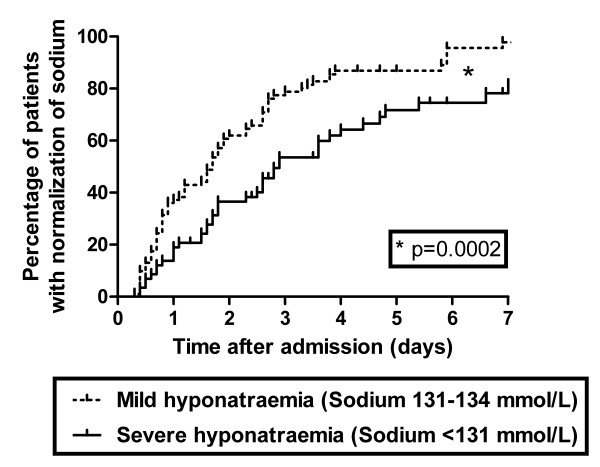
**Kaplan-Meier survival curve of restoration of hyponatraemia throughout hospitalization for malaria**. Separate curves for patients with mild and moderate-to-severe (labelled as "severe") hyponatraemia on admission are given. Mantel-Cox log-rank test showed a significant difference between the two curves (*p *= 0.002).

In a subset of 151 malaria patients with hyponatraemia from the Rotterdam Malaria Cohort, serum sodium levels were measured consecutively during hospitalization. Fifty-eight patients had moderate-to-severe hyponatraemia (16 with severe *P. falciparum *malaria; 38 patients with uncomplicated *P. falciparum *malaria; four non-falciparum malaria) and hyponatraemia was mild in 93 patients (11 patients with severe *P. falciparum *malaria; 76 with uncomplicated *P. falciparum *infection and six non-falciparum infections). As shown in Figure 1, time to normalization of serum sodium was significantly longer in patients with moderate-to-severe hyponatraemia (median time to normalization of sodium 2.9 days) than in patients with mild hyponatraemia on admission (median time to normalization of sodium 1.7 days, *p *< 0.001). In approximately 20% of the malaria patients with moderate-to-severe hyponatraemia, serum sodium levels did not normalize after one week of antimalarial treatment and infusion of isotonic saline.

### Evaluation of the role of copeptin in the pathophysiology of hyponatraemia in imported malaria

**Figure 2:**
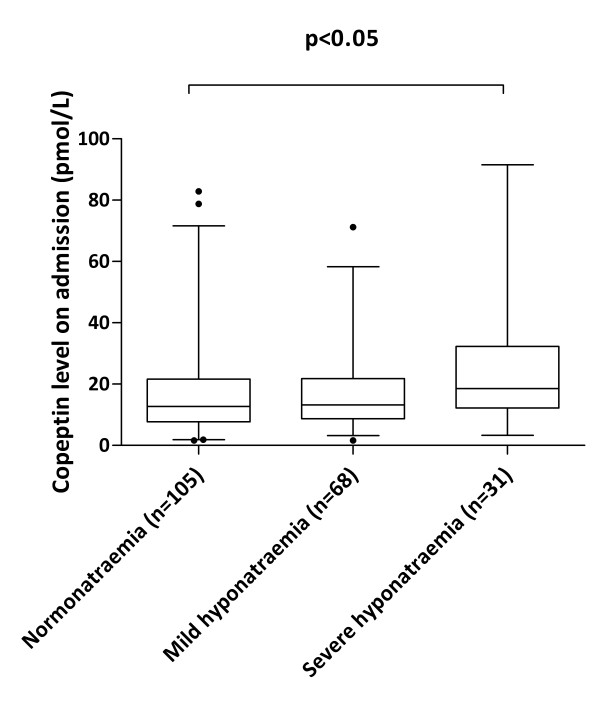
**Box- and whiskerplot of copeptin levels in malaria patients with a normal serum sodium concentration on admission (n = 105) and in patients with mild hyponatraemia (n = 68) and moderate-to-severe (labelled as "severe") hyponatraemia (n = 31) on admission**. The box indicates the lower and upper quartiles and the central line represents the median; the end of the whiskers reflect the 2.5th en 97.5th percentile of copeptin in malaria patients.

**Figure 3:**
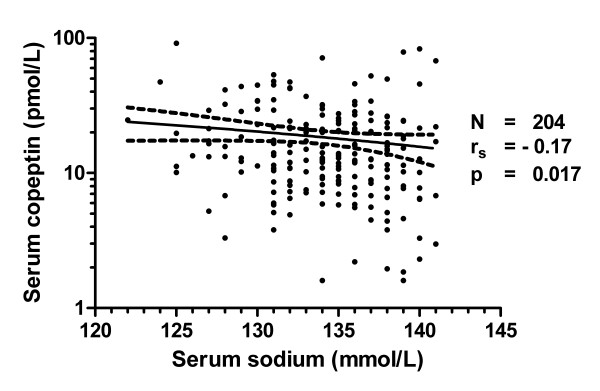
**Dot plot of relationship between serum copeptin and serum sodium**. A weak but significant inverse relationship was observed (r_s _= -0.17, *p *= 0.017).

In 204 malaria patients from the Rotterdam Malaria Cohort copeptin was measured in stored serum samples and related to previously established laboratory parameters on admission. The general characteristics of the patients participating in the copeptin study are shown in Table 1. In general, patients with moderate-to-severe hyponatraemia presented more ill, as illustrated by their higher body temperatures, pulse rates, CRP and creatinine levels. Elevated creatinine levels were frequently observed: in 12 of 31 (38.7%) patients with moderate-to-severe hyponatraemia, in 10 of 68 (14.7%) patients with mild hyponatraemia and in 20 of 105 (19.0%) of normonatraemic patients, respectively. Only two patients with severe *P. falciparum *malaria had a creatinine level exceeding the WHO threshold of 265 μmol/L for renal impairment [12]. These patients both presented with moderate-to-severe hyponatraemia on admission. Serum copeptin levels on admission were significantly higher in patients with moderate-to-severe hyponatraemia (median 18.5 pmol/L) as compared with normonatraemic patients (12.7 pmol/L) but not with malaria patients who presented with mild hyponatraemia (13.2 pmol/L, Figure 2). Copeptin levels exceeding the 97.5 percentile of normal healthy individuals (corresponding to a copeptin level of 11.25 pmol/L) were significantly (*p *= 0.0099, Chi square test for trend) more often observed in malaria patients presenting with moderate-to-severe hyponatraemia (25 of 31 [81%] patients) than in patients with patients with mild hyponatraemia (47 of 68 [69%] patients) and normonatraemia (60 of 105 [57%] patients). A poor correlation was found between serum sodium and copeptin levels on admission (Figure 3, r_s _= -0.17, *p *= 0.017). In contrast, stronger correlations were demonstrated between CRP and copeptin (Additional file 1 r_s _= -0.36, *p *< 0.0001) on the one hand and between CRP and sodium (Additional file 2 r_s _= 0.33, *p *< 0.0001) on the other hand.

**Table 1:** Characteristics of 204 malaria patients in the copeptin study.

	Moderate-to-severe hyponatraemia (n = 31)	Mild hyponatraemia (n = 68)	Normonatraemia (n = 105)	P-value*
**Demographics**				
Age, years	**42 (11-64)**	**40 (13-69)**	**39 (8 - 70)**	**n.s**.
Male, female, n (%)	**22 (71), 9 (29)**	**52 (76), 16 (24)**	**77 (73), 28 (27)**	**n.s**.
**Malaria species**				
falciparum, non-falciparum, n (%)	**26 (84), 5 (16)**	**54 (79), 12 (21)**	**61 (58), 44 (42)**	**0.0008**
Severe malaria, n (%)	**10 (40)**	**10 (40)**	**5 (20)**	**< 0.0001**
Parasite load^#^, parasites/μL	**85900 (400 - 567000) ^B < 0,001^**	**11032 (2 - 860000)**	**4600 (30 - 1380600)**	**0.0013**
**Vital signs on admission**				
Body temperature, °C	**39.0 (35.7 - 40.8)**	**38.9 (35.7 - 41.2)**	**38.2 (36.0 - 41.2)**	**0.0315**
Pulse rate, beats per minute	**100 (58 - 140 ) ^B < 0,01^**	**95 (64 - 130)**	**85 (60 - 130)**	**0.0109**
Systolic blood pressure, mm Hg	**120 (80 - 147)**	**120 (88 - 165)**	**120 (95 - 196)**	**n.s**.
**Laboratory data on admission**				
C-reactive protein, mg/L	**159 (32 - 352) ^A < 0,01; B < 0,001^**	**101 (7 - 310) ^C < 0,05^**	**78 (7 - 407)**	**< 0.0001**
Haematocrit, L/L	**0.35 (0.15 - 0.50) ^A < 0,01^**	**0.41 (0.12 - 0.52)**	**0.39 (0.26 - 0.53)**	**0.0006**
Serum glucose, mmol/L	**6.9 (4.1 - 26.0) ^B < 0,05^**	**7.0 (4.2 - 10.3) ^C < 0,001^**	**6.3 (4.1 - 14.9)**	**0.0003**
Serum creatinine, μmol/L	**111 (70 - 1081) ^B < 0,05^**	**97 (55 - 135)**	**93 (46 - 208)**	**0.0180**
Serum urea, mmol/L	**6.4 (3.6 - 55.8) ^B < 0,01^**	**5.2 (2.2 - 13.5)**	**4.9 (2.7 - 21.1)**	**0.0061**
Prerenal azotaemia^&^, n (%)	**2 (6)**	**2 (3)**	**2 (2)**	**n.s**.
Copeptin, pmol/L	**18.5 (3.3 - 91.5) ^B < 0,05^**	**13.2 (1.6 - 71.2)**	**12.7 (1.6 - 82.9)**	**0.0268**
**Duration hospitalisation**, days	**6 (1 - 13) ^B < 0,001^**	**5 (0 - 11) ^C < 0,001^**	**3 (0 - 12)**	**< 0.0001**

Parameters at initial presentation are shown in relation to serum sodium level on admission. Data are given as median (range) or as indicated otherwise.

* Univariate comparison were performed using Kruskall-Wallis (P-values are given in the column) and Dunn's post-hoc tests (p-values in superscript). A: comparison of severe hyponatraemia *vs *mild hyponatraemia; B: comparison of severe hyponatraemia *vs *normonatraemia; C: comparison of mild hyponatraemia *vs *normonatraemia. ^# ^P.falciparum parasite load only. ^&^defined as ratio serum urea/serum creatinin > 1:10; n.s. = denotes "not significant difference"

### Urine biochemistry

In a subset of 23 malaria patients urine sodium and urine osmolality were measured in parallel with measurement of serum copeptin. The main outcome measures are shown in Table 2. In none of the 13 hyponatraemic patients, serum glucose exceeded 10 mmol/L, excluding hyperglycaemia as a significant cause of hyponatraemia. When the 13 hyponatraemic malaria patients were categorized according to the serum copeptin to urine sodium ratio, 7 hyponatraemic patients fulfilled the criteria for inappropriate AVP secretion. Median copeptin levels in patients with appropriate AVP release were twofold higher than in patients with inappropriate AVP release (23.5 *vs *11.4 pmol/L, Table 2). There were no significant differences in creatinine, haematocrit or urea to creatinine ratio between hyponatraemic malaria patients with inappropriate or appropriate AVP secretion, even though four patients with appropriate AVP secretion had a creatinine level above the normal range (two of them fulfilled the WHO criterion for renal impairment). All patients with inappropriate AVP secretion had creatinine levels within the normal range. Urine osmolality values were significantly higher in patients with inappropriate AVP release (median 780 mOsm/kg) than in patients with appropriate AVP release (480 mOsm/kg) or normonatraemic patients (484 mOsm/kg). Patients with inappropriate AVP release also had significantly higher body temperatures on admission than hyponatraemic patients with appropriate AVP release.

**Table 2:** Results of parallel measurements of urine and blood samples from hyponatraemic and normonatraemic malaria patients on admission.

Parameter	Hyponatraemic patients (n = 13)		Normonatraemic patients (n = 10)		
	**Inappropriate AVP secretion^@^** **(n = 7)**	**Appropriate AVP secretion** **(n = 6)**	**P-value#**		P-value*

**Vital signs on admission**					

Body temperature, °C	38.9 (37.6-41.1)	37.4 (35.7-38.6)	P = 0.0153	38.3 (36.0-40.1)	n.s.

Pulse rate, beats per minute	96 (72-121)	105 (91-120)	n.s.	93 (72-125)	n.s.

**Laboratory data on admission**					

C-reactive protein, mg/L	158 (60-176)	236 (71-352)	n.s.	95 (18-407)	n.s.

Haematocrit, L/L	0.39 (0.15-0.44)	0.40 (0.19-0.50)	n.s.	0.46 (0.36-0.51)	n.s.

Serum Urea:creatinine ratio	0.06 (0.04-0.11)	0.07 (0.05-0.14)	n.s.	0.06 (0.03-0.09)	n.s.

Serum copeptin, pmol/L	11.4 (7.2 -21.4 )	23.5 (6.8 -91.5 )	n.a.	12.2 (3.8-49.7)	n.a.

Serum copeptin > P97.5, n (%)	4 (57)	5 (83)	n.s.	6 (60)	n.s

Serum sodium, mmol/L	132 (131-134)	128 (124-132)	P = 0.012	138 (135-141)	n.a.

Urine osmol, mosmol/kg	780 (540-924)	480 (298-532)	P = 0.0022	484 (234-906)	P = 0.047

Urine sodium, mmol/L	49 (38-154)	9 (9-47)	n.a.	32 (9-164)	n.a.

Data are given as median (range) or as indicated otherwise.

Legend to the table: @ = inappropriate AVP secretion was defined as a serum copeptin to urine sodium ratio of ≤ 30 pmol/mmol. #P-values of comparison of hyponatraemic patients with inappropriate vs appropriate AVP secretion. *P-values of univariate analysis using Kruskall Wallis followed by Dunn's post hoc tests; n.s. = not significant difference; n.a. = not applicable (defining criterion).

## Discussion

Copeptin, the C-terminal glycopeptide domain of pro-vasopressin, is co-secreted with AVP from the posterior pituitary in hyperosmolar states and upon multiple non-osmotic stimuli, such as hypotension, pain, and other non-specific causes of stress [6,8]. Circulating copeptin levels are therefore thought to reflect the activity of the neuroendocrine stress axis. To gain more insight in the pathophysiology of hyponatraemia in malaria and in particular the role of AVP, serum copeptin was measured in a large cohort of 204 patients with imported malaria. In malaria patients the median serum copeptin levels were three to five-fold higher than the median level of 4.2 pmol/L observed in 359 healthy volunteers [8]. In fact, the proportion of malaria patients with copeptin levels above the 97.5th percentile of normal significantly increased with decreasing sodium levels (Figure 3). Moreover, in absolute terms, patients with moderate-to-severe hyponatraemia also had significantly higher copeptin levels than normonatraemic malaria patients on admission (Figure 2). Because the physiological stimulus for AVP release is hypertonicity, elevated AVP or copeptin levels in the context of hyponatraemia indicate a pathological setting. That is, during normal physiology, the development of hyponatraemia ought to suppress AVP release and to result in a maximally dilute urine with a low urine osmolality [5,17].

A recent study found that the ratio of serum copeptin to urine sodium may be used to differentiate inappropriate from appropriate AVP secretion [10]. To further investigate the antidiuretic effect of AVP at the level of the target organ, urine sodium and osmolality were prospectively studied in parallel with measurements of serum copeptin levels in a subset of 13 hyponatraemic and 10 normonatraemic malaria patients on admission. Based on the serum copeptin to urine sodium ratio, six patients had appropriate AVP release, while AVP release was inappropriate in seven patients. Hyponatraemic patients with inappropriate AVP release had significantly higher urine osmolality values than observed in patients with an appropriate AVP response or in normonatraemic patients. This suggests active water reabsorption by the kidneys in malaria patients with inappropriate AVP release. Why inappropriate AVP release results in a higher urine osmolality than appropriate AVP release is unclear. One could speculate that in the group with appropriate AVP release, the renin angiotensin system was likely also activated, leading to increased renal sodium reabsorption. Because urine sodium is a major determinant of urine osmolality, a lower urine sodium would, therefore, result in a lower urine osmolality in malaria patients with appropriate AVP release.

From a pathophysiological point of view there may be two possible explanations for the increased serum copeptin levels despite the presence of hypotonicity. First, volume regulation may have overruled osmoregulation if there was true hypovolaemia [18] or a low effective arterial blood volume [19]. This mechanism is mediated via baroreceptors in the vasculature and is often referred to as "appropriate" AVP release. In 6 of 13 hyponatraemic patients with available urine biochemistry data AVP release was considered appropriate based on the serum copeptin to urine sodium ratio. However, in a substantial number of patients with moderate-to-severe hyponatraemia, the hyponatraemia persisted for more than 7 days despite infusion of isotonic saline, rendering persistence of hypovolaemia an unlikely explanation (Figure 1). Hence, other mechanisms must apply in a substantial number of malaria patients with hyponatraemia.

The second explanation for elevated copeptin levels despite the presence of hypotonicity may involve activation of central osmoreceptors leading to vasopressin release and subsequent development of hyponatraemia. This alternative mechanism could have been mediated through cytokines [20] and resembles the syndrome of inappropriate antidiuresis, a common cause of hyponatraemia [15]. In fact, in seven of 13 hyponatraemic malaria patients an inappropriate release of AVP appeared to be present. Of potential relevance, in this regard, is the observation that the pro-inflammatory cytokine interleukin-6 is elevated in malaria and also implicated in the non-osmotic release of AVP [21,22]. The delayed normalization of serum sodium concentration, as was observed in the present study, might be the consequence of the persistent elevation of pro-inflammatory cytokines, as has been shown for patients with severe malaria [23]. Previously, a relationship between a rise in CRP and the development of in-hospital hyponatraemia was demonstrated [24]. This is not only another illustration of a presumed cytokine-driven non-osmotic release of AVP [20] but also in line with the observed inverse relationship between serum sodium and CRP levels on the one hand and CRP and copeptin levels on the other hand (Additional files 1 and 2). Although several drugs, such as opiates, non-steroidal anti-inflammatory drugs, and diuretics, can contribute to hyponatraemia, these drugs were rarely used in this cohort, and it is common policy not to administer these drugs to malaria patients because of their potentially adverse effects. Although thyroid and adrenal function were not formally assessed, which is recommended before diagnosing inappropriate AVP release, the response of hyponatraemia to malaria treatment was highly suggestive of a causal relationship.

The distinction between appropriate and inappropriate AVP release in hyponatraemic malaria patients may be relevant with regard to selecting the optimal intravenous fluid regimen. Because previous studies did not separate hyponatraemic malaria patients on the basis of appropriate or inappropriate AVP secretion, future studies are necessary to give clinical guidance. In general, however, hypovolaemia causes appropriate AVP release and should therefore be treated with isotonic fluids. A caveat, however, is that serum sodium may rise too rapidly during treatment of hypovolaemic hyponatraemia with isotonic fluids [25]. The risk of exceeding recommended correction rates is osmotic demyelination, although few cases in malaria patients have been reported. Conversely, during inappropriate AVP release, the emphasis of therapy should perhaps be more on aggressive anti-malaria treatment, given the association with a stronger pro-inflammatory cytokine response. In this setting, a restrictive intravenous fluid regimen may prove beneficial, because even isotonic saline can worsen hyponatraemia during the syndrome of inappropriate antidiuresis [26]. In this regard, a recent study advocating restrictive IV-fluid therapy in children with malaria is also of interest, although no serum sodium values were reported [27].

A potential limitation of our study is that it remains debatable whether urine sodium can be considered a reliable parameter for the establishment of hypovolaemia in malaria, since circulating cytokines have also been incriminated in causing tubular injury and therefore natriuresis [28]. The evidence for the pathogenetic role of AVP in the pathophysiology of hyponatraemia in malaria is substantial. The results of the small urine substudy suggest that appropriate [2,19] and inappropriate [3,29] AVP secretion may both occur in the pathophysiology of hyponatraemia in imported malaria. However, the high proportion of patients with appropriate AVP release who had elevated creatinine levels on admission, combined with the higher pulse rate, haematocrit, serum urea to creatinine ratio and the twofold increase in median AVP release as compared with patients with inappropriate AVP secretion, are all in support of a hypovolaemia-driven release of AVP. Although speculative, the significantly higher body temperatures observed in patients with inappropriate AVP release on admission suggests that - at least in part - the extent of the host inflammatory response to the invading malaria parasite may play a pivotal role in the aetiology of cytokine-driven non-osmotic release of AVP.

## Competing interests

The authors declare that they have no competing interests.

## Authors' contributions

EJH contributed to the data analysis and writing of the manuscript. MEvW, DAH and JvH participated in the data analysis and revising of the manuscript. YdeR carried out the copeptin measurements and contributed to the data analysis. RK is responsible for collection of patient materials and database management. PJvG participated in the data acquisition and analysis and in writing and revising the manuscript. All authors have seen and approved the final version.

## Supplementary Material

Additional file 1 **Dot plot of relationship between serum C-reactive protein and serum Copeptin on admission as a function of sodium level on admission**. A significant correlation between C-reactive protein and serum Copeptin was present (r_S _= 0.33, *p *< 0.0001). Patients with moderate or severe hyponatraemia were grouped (labelled as "severe"). Click here for file

Additional file 2 **Dot plot of relationship between serum C-reactive protein and serum sodium on admission as a function of copeptin on admission**. Copeptin levels above and below the 97.5th percentile of normal are separately given. A significant inverse correlation was present between C-reactive protein and sodium on admission (r_S _= -0.36, *p *< 0.0001). Click here for file

## References

[B1] van Wolfswinkel ME Hesselink DA Zietse R Hoorn EJ van Genderen PJ Hyponatraemia in imported malaria is common and associated with disease severity Malar J 2010 25 140 10.1186/1475-2875-9-140 PMC2890675 20497587

[B2] Hanson J Hossain A Charunwatthana P Hassan MU Davis TM Lam SW Chubb SA Maude RJ Yunus EB Haque G White NJ Day NP Dondorp AM Hyponatremia in severe malaria: evidence for an appropriate anti-diuretic hormone response to hypovolemia Am J Trop Med Hyg 2009 80 141 145 19141852 10.4269/ajtmh.2009.08-0393 PMC2843441

[B3] Holst FG Hemmer CJ Kern P Dietrich M Inappropriate secretion of antidiuretic hormone and hyponatremia in severe falciparum malaria Am J Trop Med Hyg 1994 50 602 607 8203710 10.4269/ajtmh.1994.50.602

[B4] Miller LH Makaranond P Sitprija V Suebsanguan C Canfield CJ Hyponatraemia in malaria Ann Trop Med Parasitol 1967 61 265 279 4867548 10.1080/00034983.1967.11686487

[B5] Robertson GL Antidiuretic hormone. Normal and disordered function Endocrinol Metab Clin North Am 2001 30 671 694 vii 10.1016/S0889-8529(05)70207-3 11571936

[B6] Jochberger S Morgenthaler NG Mayr VD Luckner G Wenzel V Ulmer H Schwarz S Hasibeder WR Friesenecker BE Dünser MW Copeptin and arginine vasopressin concentrations in critically ill patients J Clin Endocrinol Metab 2006 91 4381 4386 10.1210/jc.2005-2830 16940457

[B7] Struck J Morgenthaler NG Bergmann A Copeptin, a stable peptide derived from the vasopressin precursor, is elevated in serum of sepsis patients Peptides 2005 26 2500 2504 10.1016/j.peptides.2005.04.019 15922490

[B8] Morgenthaler NG Struck J Jochberger S Dunser MW Copeptin: clinical use of a new biomarker Trends Endocrinol Metabol 2007 19 43 49 10.1016/j.tem.2007.11.001 18291667

[B9] Morgenthaler NG Struck J Alonso C Bergmann A Assay for the measurement of copeptin, a stable peptide derived from the precursor of vasopressin Clin Chem 2006 52 112 119 10.1373/clinchem.2005.060038 16269513

[B10] Fenske W Störk S Blechschmidt A Maier SG Morgenthaler NG Allolio B Copeptin in the differential diagnosis of hyponatremia J Clin Endocrinol Metab 2009 94 123 129 18984663 10.1210/jc.2008-1426

[B11] Fenske W Quinkler M Lorenz D Zopf K Haagen U Papassotiriou J Pfeiffer AF Fassnacht M Stork S Allolio B Copeptin in the differential diagnosis of the polydipsia-polyuria syndrome - revisiting the direct and indirect water deprivation tests J Clin Endocrinol Metab 2011 96 1506 1515 10.1210/jc.2010-2345 21367924

[B12] Guidelines for the treatment of malaria World Health Organization 2010 Second http://www.who.int/malaria/publications/atoz/9789241547925/en/index.html 25473692

[B13] Van Genderen PJ van der Meer IM Consten J Petit PL van Gool T Overbosch D Evaluation of plasma lactate as a parameter for disease severity on admission in travelers with *Plasmodium falciparu *malaria J Travel Med 2005 12 261 264 16256049 10.2310/7060.2005.12504

[B14] Hanson J Lee SJ Mohanty S Faiz MA Anstey NM Charunwatthana P Yunus EB Mishra SK Tjitra E Price RN Rahman R Nosten F Htut Y Hoque G Chau TTH Phu NH Hien TT White NJ Day NPJ Dondorp AM A simple score to predict the outcome of severe malaria in adults Clin Infect Dis 2010 50 679 685 10.1086/649928 20105074 PMC4313369

[B15] Ellison DH Berl T Clinical practice. The syndrome of inappropriate antidiuresis N Engl J Med 2007 356 2064 2072 10.1056/NEJMcp066837 17507705

[B16] van Genderen PJ Hesselink DA Bezemer JM Wismans PJ Overbosch D Efficacy and safety of exchange transfusion as an adjunct therapy for severe Plasmodium falciparum malaria in nonimmune travelers: a 10-year single-center experience with a standardized treatment protocol Transfusion 2010 50 787 794 10.1111/j.1537-2995.2009.02488.x 19951317

[B17] Hoorn EJ Zietse R Hyponatremia revisited: translating physiology to practice Nephron Physiol 2008 108 46 59 10.1159/000119709 18319606

[B18] Dunn FL Brennan TJ Nelson AE Robertson GL The role of blood osmolality and volume in regulating vasopressin secretion in the rat J Clin Invest 1973 52 3212 3219 10.1172/JCI107521 4750450 PMC302597

[B19] Sitprija V Napathorn S Laorpatanaskul S Suithichaiyakul T Moollaor P Suwangool P Sridama V Thamaree S Tankeyoon M Renal and systemic hemodynamics, in falciparum malaria Am J Nephrol 1996 16 513 519 10.1159/000169042 8955763

[B20] Swart RM Hoorn EJ Betjes MG Zietse R Hyponatremia and Inflammation: the emerging role of interleukin-6 in osmoregulation Nephron Physiol 2011 118 45 51 21196778 10.1159/000322238

[B21] Mastorakos G Weber JS Magiakou MA Gunn H Chrousos GP Hypothalamic-pituitary-adrenal axis activation and stimulation of systemic vasopressin secretion by recombinant interleukin-6 in humans: potential implications for the syndrome of inappropriate vasopressin secretion J Clin Endocrinol Metab 1994 79 934 939 10.1210/jc.79.4.934 7962300

[B22] Palin K Moreau ML Sauvant J Orcel H Nadjar A Duvoid-Guillou A Dudit J Rabié A Moos F Interleukin-6 activates arginine vasopressin neurons in the supraoptic nucleus during immune challenge in rats Am J Physiol Endocrinol Metab 2009 296 e1289 1299 10.1152/ajpendo.90489.2008 19258490

[B23] Ballal A Saeed A Rouina P Jelkmann W Effects of chloroquine treatment on circulating erythropoietin and inflammatory cytokines in acute Plasmodium falciparum malaria Ann Hematol 2009 88 411 415 10.1007/s00277-008-0636-z 19031076

[B24] Beukhof CM Hoorn EJ Lindemans J Zietse R Novel risk factors for hospital-acquired hyponatraemia: a matched case-control study Clin Endocrinol (Oxf) 2007 66 367 372 10.1111/j.1365-2265.2007.02741.x 17302870

[B25] Liamis G Kalogirou M Saugos V Elisaf M Therapeutic approach in patients with dysnatraemias Nephrol Dial Transplant 2006 21 1564 1569 10.1093/ndt/gfk090 16449285

[B26] Steele A Gowrishankar M Abrahamson S Mazer CD Feldman RD Halperin ML Postoperative hyponatremia despite near-isotonic saline infusion: a phenomenon of desalination Ann Intern Med 1997 126 20 25 8992919 10.7326/0003-4819-126-1-199701010-00003

[B27] Maitland K Kiguli S Opoka RO Engoru C Olupot-Olupot P Akech SO Nyeko R Mtove G Reyburn H Lang T Brent B Evans JA Tibenderana JK Crawley J Russell EC Levin M Babiker AG Gibb DM Mortality after fluid bolus in African children with severe infection N Engl J Med 2011 364 2483 2495 10.1056/NEJMoa1101549 21615299

[B28] Schmidt C Hocherl K Schweda F Bucher M Proinflammatory cytokines cause down-regulation of renal chloride entry pathways during sepsis Crit Care Med 2007 35 2110 2119 10.1097/01.ccm.0000281447.22966.8b 17855824

[B29] Sowunmi A Newton CR Waruiru C Lightman S Dunger DB Arginine vasopressin secretion in Kenyan children with severe malaria J Trop Pediatr 2000 46 195 199 10.1093/tropej/46.4.195 10996978

