# Supplementary figures and images for: Hyponatraemia in imported malaria: the pathophysiological role of vasopressin
Source: Malar J. 2012 Jan 26;11:26. doi: 10.1186/1475-2875-11-26 (PMC3296600; doi:10.1186/1475-2875-11-26)

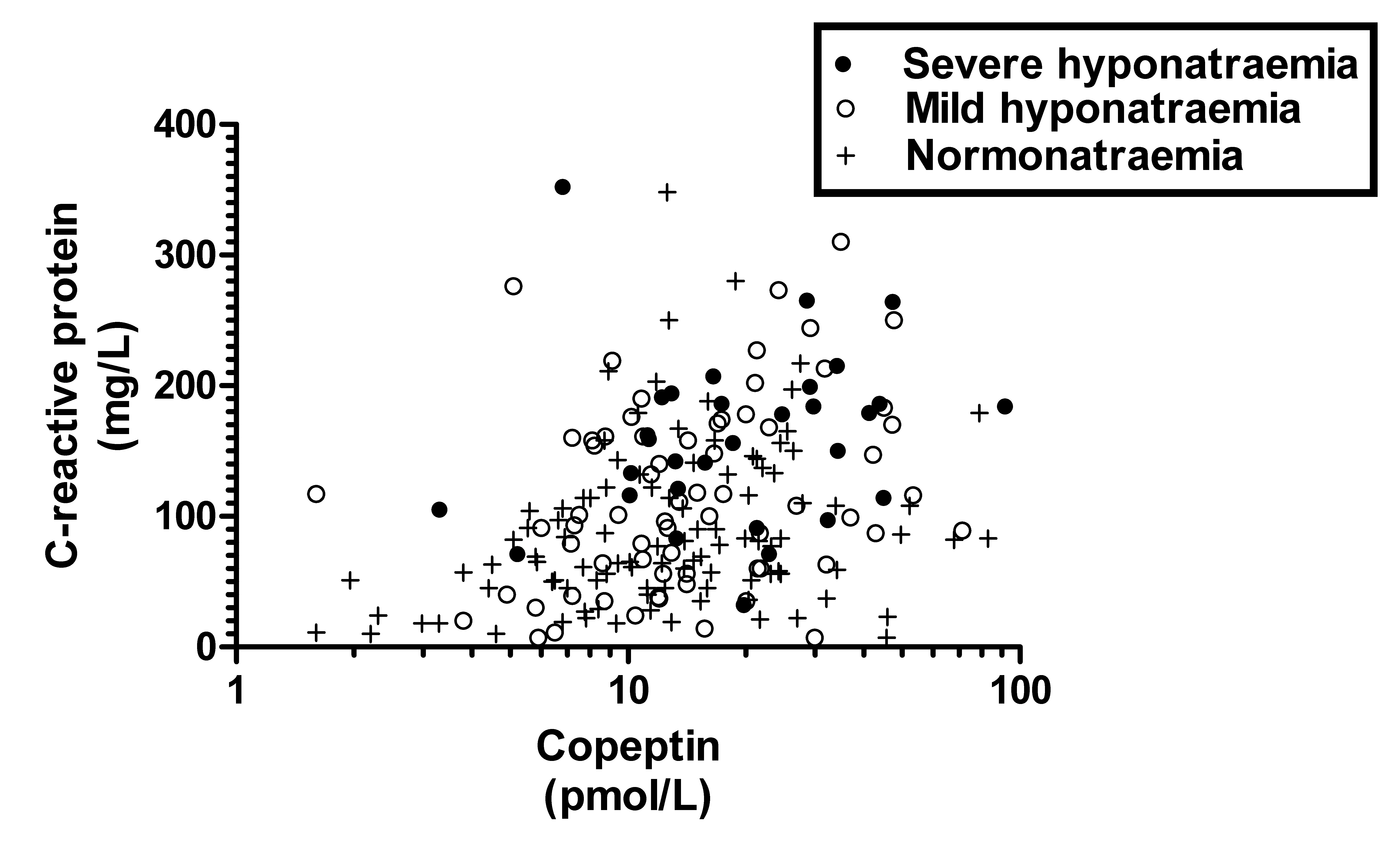

Supplement: Additional file 1 — Dot plot of relationship between serum C-reactive protein and serum Copeptin on admission as a function of sodium level on admission. A significant correlation between C-reactive protein and serum Copeptin was present (rS = 0.33, p < 0.0001). Patients with moderate or severe hyponatraemia were grouped (labelled as "severe"). [file 1475-2875-11-26-S1.JPEG]

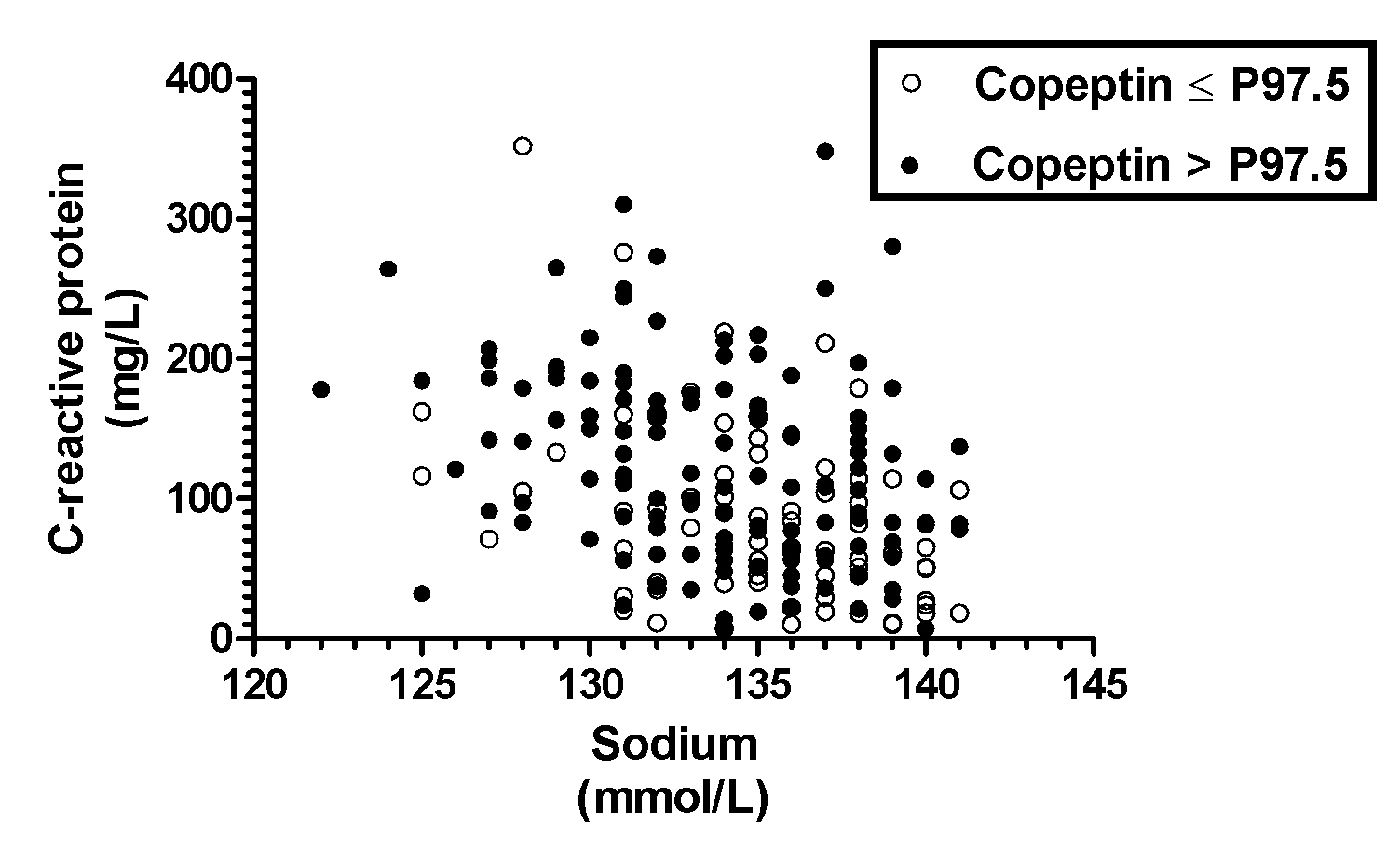

Supplement: Additional file 2 — Dot plot of relationship between serum C-reactive protein and serum sodium on admission as a function of copeptin on admission. Copeptin levels above and below the 97.5th percentile of normal are separately given. A significant inverse correlation was present between C-reactive protein and sodium on admission (rS = -0.36, p < 0.0001). [file 1475-2875-11-26-S2.JPEG]
